# Medicine

**Published:** 1913-09

**Authors:** George Parker


### Progress of the Medical Sciences.

### MEDICINE.

The old questions as to the importance of abnormally **high blood pressures** and as to whether they are compensatory, or part of a vicious circle, or merely a by-product of disease, have been of late the subject of many dogmatic statements as well as of some important researches. T. Janeway[1] has reviewed in detail the abundant work done of late on hypertension in kidney disease, and has to confess that neither epinephrin nor anything else has been shown to be the definite agent. His own view is clearly in favour of the old theory of arterio-capillary fibrosis preceding actual kidney disease. In another paper[2] he attacks the question by a fresh method, and tabulates the causes of death in 100 cases of high pressure, *i.e.* of 170 mm.

[1] *Am. J. M. Sc.*, 1913, cxlv. 1. [2] *J. Am. M. Ass.*, 1912, lix, 2106.

and upwards. Among his 100 patients 79 had been diagnosed as suffering from some form of nephritis, 7 from the diabetes of elderly people, 9 from heart affections, and 5 from arterio-sclerosis or from simple high pressure. Now, death occurred in 35 from uræmia, and from some form of circulatory trouble in 50, that is to say in 29 from cardiac failure, in 3 from angina, in 14 from cerebral apoplexy, and in 4 from acute œdema of the lungs, while in 13 instances the cause of death was unknown or unconnected with the history. Thus uræmia accounted for only a comparatively small group, one-third of the whole, and in them Janeway notes that the most frequent symptoms had been polyuria, headaches, and disturbances of vision. On the other hand, some breakdown in the heart or vessels took place in a rather larger group equal to half the whole number. Here the earliest prominent symptoms had been dyspnœa on exertion. The actual deaths from angina were very few. Those from cerebral hemorrhage were only one-seventh of the whole, but we do not learn how many patients were crippled by non-fatal hemorrhagic hemiplegia. In any case, the total of these hemorrhages must have been large and the danger a real one. It may be argued from these figures that (1) where the compensatory pressure was insufficient death took place from uræmia, *i.e.* in a third of the cases ; (2) where the pressure was sufficient death occurred from failure of the heart or vessels, *i.e.* in at least half of the cases. However, the theory of compensatory high pressure is a doubtful one. Again, considering the large number of patients originally diagnosed as nephritics, the question arises whether in many of them the nephritis was not altogether secondary to arterio-sclerosis. Osler thinks that it is not difficult to distinguish primary nephritis from primary sclerosis, but probably mistakes are often made about it. It is much to be wished that others would continue Janeway's work, and investigate the end of some thousands of these cases, so that more reliable indications for treatment might be obtained.

C. H. Lawrence,[1] at the Massachusetts General Hospital, has carried out most elaborate investigations to settle the question whether increased blood pressure does or does not cause an increased secretion of urine. In reporting them, he points out that while it is asserted that high blood pressures are protective, it is a curious fact that most of the ordinary drugs found useful in nephritis are vaso-depressants ; and again, that Senator and others have fully proved in animals (1) that high blood pressure is not produced by any obliteration of or artificially increased resistance in the kidney vessels, (2) nor does it increase the supply of blood to the kidney, (3) nor the urine excreted. Since a certain amount of clinical evidence

[1] *Am. J. M. Sc.*, 1912, cxliv. 330.

opposed to this had been brought forward with respect to human beings, Lawrence obtained 20 patients who had each a systolic pressure over 180, and by constant and detailed investigations under minute precautions against error obtained 627 observations on changes in blood pressure which coincided with changes in the amount of urine. These and subsequent tests showed that—

(1) There is no clear connection between changes in the systolic or in the diastolic pressure and the amount of urine excreted.

(2) An increase does indeed occur when the "pulse pressure" increases with a falling systolic pressure.

(3) There is also an increase when the administration of a nitrite increases the output of blood from the heart.

Thus high pressure is not protective, for it has little or no influence on the blood supply of the kidney or on the excretion of urine. However, the amount of blood passing through the kidney in a unit of time is important.

In connection with these results, it is curious to note the remarkable evidence recently brought forward as to the action of digitalis by F. W. Price, J. Burnett and others. Digitalis has long been credited with a diuretic action and with causing a rise in blood pressure. Thus it has often been withheld where a rise of pressure would be dangerous. However much a heart stood in need of digitalis, it seemed almost criminal to give it if the peripheral pressure was very high. Price,[1] however, conducted a series of most carefully-guarded observations upon a number of patients, and only once in 21 cases was there any rise of pressure, whilst in many of them there was an actual fall. Burnett[2] got similar results in 25 cases, including both healthy and diseased persons, and we may add that the doses employed in the experiments varied from 45 to 90 minims of the tincture daily. Thus there is strong direct evidence that in man digitalis does not raise pressure by constriction of the vessels. Josué and Godlewsky also report to the Société Médicale des Hôpitaux that they find digitalis does not raise the pressure either in patients with a normal tension or in arterio-sclerotics whose tension is already high, though they hold that it may do so in hypotension with cardiac insufficiency.

Herbert French[3] argues that we need not imagine various retained toxins causing peripheral constriction. When the muscular coats of, say, the gastric or splanchnic area are kept permanently dilated they become fibrotic, just as the muscles of an arm which is kept fixed in one position for a long period. These sclerosed vessels cannot contract or dilate, and if an increased blood supply is required for any area it can only be

[1] *Brit. M. J.*, 1912, ii. 689.

[2] *Med. Press and Circ.*, 1912, cxlv. 182. [3] *Lancet*, 1912, ii. 850.

obtained by forcing the blood into all vessels at a higher pressure. Here, again, we have the compensatory theory postulated, whereas the existence of fibrosis is sufficient cause for the rise of pressure if the heart remains vigorous.

**Radium Emanation.**—Certain definite facts as to the physiology and effects of treatment by Radium Emanation or Niton have been gradually made clear, whereas a year or two ago much discredit on its use was brought about by the unwarranted claims put forward, by the uncertainty as to dosage, and by the absurdly weak emanations employed. Even Gudzent's experiments on gout and his statements as to the effect of the Emanation on the excretion of uric acid were contradicted; but a worker in Madame Curie's laboratory has confirmed his results, and has shown that the Emanation does change the insoluble urates into more soluble ones, greatly increases the excretion of purin bodies and of endogenous and exogenous uric acid. No one now denies certain effects of the X-rays, such as their influence in skin diseases and on the blood cells, or the dangers of their use; so, too, the laboratory tests of the action of the Emanation and of Thorium X give definite physical results. Thus there is evidence of their action on pepsin, pancreatin, and the autolytic ferments, on phagocytosis, on yeast and lactic acid fermentations, on coagulation of the blood, and on the life of certain bacteria (Engelman,[1] Van der Velde, and others). Bellingham Smith[2] shows that elimination of the Emanation takes place chiefly through the lungs, and is complete in about four hours, in small mammals, prior to which it causes general radio-activity throughout the body. Bodies which affect metabolism so markedly should clearly not be used without discrimination, and Carl von Noorden, who by the help of the Austrian Government employs in his clinic radio-active water to the value of £8,000 or £10,000 a year, lays down that they must not be used (1) where there is a tendency to any kind of hemorrhage, (2) in severe forms of diabetes, Graves' disease, and in advanced blood disorders, (3) in febrile complaints, or (4) in marked cardiac weakness.[3]

Indeed, Thorium X, which produces most striking improvement in blood diseases, and has the advantage of being cheap and easily measured, has already caused a fatal accident to a patient with some hemorrhagic affection at Berlin. Among clinical results von Noorden notices (1) the increase of respiratory metabolism by 27 per cent., and also of carbohydrate metabolism, which was confirmed by Kikkoji and Bernstein; (2) a great increase both of red and white blood cells, except in diseases where the power of regeneration was exhausted, or where the dose of Emanation was too great, in which case a

[1] *Med. Rec.*, 1913, lxxxiii. 95.

[2] *Quart. J. Med.*, 1912, v. 249. [3] *Med. Rec.*, *loc. cit.*

diminution took place; (3) the increase of purin metabolism, so that the excretion of uric acid was sometimes actually doubled, while the pain, the swelling of joints, and neuralgias in chronic gout were notably relieved (Gudzent treated 400 cases of gout, rheumatism, and arthritis deformans with much success); (4) remarkable removal of pain and swelling, together with increased mobility in arthritis deformans and sub-acute rheumatism when full doses were used (acute rheumatism and spondylitis are probably unaffected; some observers had great success with gonorrheal rheumatism, and others failed); (5) very good results in the treatment of insomnia, nervous excitability, and in some forms of gastric irritation, vomiting and hiccough; (6) reduction of abnormally high blood pressure under the use of Thorium, but the action of the Emanation was here very uncertain. Von Noorden usually combines strongly radio-active baths with inhalations and draughts of the water.

Pagan Lowe raises the question whether artificially-made water is as efficient as the natural springs with their saline constituents.[1] Some of the clinical reactions are certainly given by the former, but whether the results are so satisfactory is an open question. However, the effects of the Buxton water, where the radio-active springs contain hardly any salines, compare well with those of other places. Armstrong claims that these waters give excellent results in gout, rheumatism, dyspepsia, and abnormally high blood pressure; for the latter he has employed with much success radio-oxygen baths. In Germany the most powerful waters are those of Gastein, Landeck, Baden-Baden, Kreuznach, Nauheim, and above all Joachimsthal, which is four times the strength of any other. In France the most active spring is Plombières. The waters here used in the celebrated Plombières douche have been most useful in mucous colitis and other disorders.

**Fibrositis.**—The gradual separation of the different diseases once comprehended under the term rheumatism have hardly yet been completed, and the name itself only seems to date from the work of Ballonius in the first half of the seventeenth century. Then Sydenham quickly distinguished between gout and rheumatism; and from about 1760 many writers under the teaching of Heberden have separated arthritis deformans from rheumatism. As early as 1816 Balfour described a fourth form, viz. muscular rheumatism. To this Gowers gave the name of fibrositis, and Stockman made clear for us its pathology. Although a monograph was written on the subject by as early as 1856, it is astonishing how scanty its literature is in this country compared to that in Sweden and Germany.

In a recent discussion[2] Llewellyn pointed out that the

[1] *Proc. Roy. Soc. Med.* 1912, v. *Baln. Sect.* 29.

[2] *Ibid.*, 1913, vi. *Baln. Sect.* 27.

essential lesions consist in an inflammatory hyperplasia and exudation in the white fibrous tissue in various parts of the body; for example, in the sheaths and attachments of muscles, in the investment of joints and bursæ and of nerve trunks. In this tissue Stockman has found new blood vessels affected with endarteritis, but whether the condition is due to the absorption of toxins or to an invasion of one or various microbes is unknown. Frequently a direct injury or exposure to cold is the immediate precursor. In muscles the spindles or sensory organs of Ruffini and Golgi are compressed by this new tissue and become hypersensitive. Besides the excruciating pains on movement during the acute stage, the tonus of the affected muscles is magnified by the excessive impulses sent up constantly from these organs, and this irritable hypertonic state lasts even after the pain, heat and swelling of the acute stage have gone away.

In the chronic types the new tissue forms nodules in the muscles or around their attachments, which with a little care can be detected through the skin. They may feel like small beads, cords, or flat plates, or rounded masses, varying much in size and hardness. In the numerous cases of brachial neuritis, so common in recent years, nothing is more striking than the number of tender points which can be found on deep pressure. There will be one over the insertion of the deltoid, one over the capsule of the joint, or, as Luff[1] suggests, over the subacromial bursa, perhaps one in the substance of the pectoralis major, and two or three over the edges of the scapula. The patient may be quite unaware of them until they are touched. The condition may spread to the neighbouring nerves or set up a neuritis in them. In the abdominal wall fibrositis is not rarely mistaken for appendicitis, biliary or renal colic, or gastric ulcer, but in these the tenderness is more marked when the muscles are relaxed, whereas, as George A. Murray[2] points out, in fibrositis there is hardly any tenderness or pain until the muscle is contracted. Often, too, a localised swelling or node can be felt, perhaps along the edge of the rectus or at its attachment to the costal cartilages. In lumbago and stiff neck similar infiltrations and nodes can be felt, and tender points when the muscles are contracted. In some cases of sciatica the nodes can be found near or along the nerve trunk. Maxwell Telling[3] lays down that many intractable headaches put down to myopia or some other refuge of the destitute are due to fibrositis in the temporal and retrocollic fascia. Luff finds this frequently in motorists. Dupuytren's contraction, tennis elbow, pains in the joints and loins after a public dinner and the absorption of various toxins and bad wines, and the rare

[1] *Brit. M. J.*, 1913, i. 757.

[2] *Clin. J.*, 1910, xxxvi. 49.

[3] *Lancet*, 1913, i. 154.

adiposis dolorosa, are according to Luff also instances of fibrositis.

In some of these forms the constant wearing pain and the violent exacerbations at times reduce the patient to a miserable state of depression as well as of incapacity for work. Treatment is often difficult. For the relief of pain aspirin either alone or with pyramidon, ammon. chloride in 20 grain doses, or phenacetin and gelsemium are often useful, but to prevent a return a full course of potassium iodide or of guaiacum resin is desirable. For local applications relief is obtained by salicylic acid and menthol in lanoline, or chloral menthol and camphor, or (Luff) a fomentation applied over tincture of iodine painted on the skin, or hot sand bags, or radiant heat followed at once by ionisation with lithium iodide. Ordinary general massage is useless, but a masseur is invaluable who knows how to pick out the nodes and tender spots, and while exceedingly gentle at first, especially with inflamed joints, bursæ and nerve sheaths, gradually removes the indurations. As long as the fibrous thickening remains a strain or chill is apt to bring on a relapse.

G. PARKER.

